# Effects of Ninjin’yoeito and physical exercise on serum corticosterone and hippocampal BDNF/proBDNF and neuroinflammation in post-stroke depression in rats

**DOI:** 10.1186/s12906-025-04915-w

**Published:** 2025-05-13

**Authors:** Harutoshi Sakakima, Nao Nojima, Akira Tani, Kazuki Nakanishi, Teruki Matsuoka, Ryoma Matsuzaki, Shogo Kakimoto, Yuki Kato, Yuta Tachibe, Masaki Inadome, Takuya Kawatani, Shotaro Otsuka, Keita Mizuno, Ikuro Maruyama

**Affiliations:** 1 Department of Physical Therapy, School of Health Sciences, Faculty of Medicine, Kagoshima University, 8-35-1, Sakuragaoka, Kagoshima, 890-8544 Japan; 2 Faculty of Welfare and Health Science, Oita University, Oita, Japan; 3 Kampo Research and Development Division, Tsumura Kampo Research Laboratories, Tsumura and Co., Ibaraki, Japan; 4 Department of Laboratory and Vascular Medicine, Graduate School of Medical and Dental Sciences, Kagoshima University, Kagoshima, Japan

**Keywords:** PSD, Ninjin’yoeito, Neurotrophic factor, Neurogenesis, Neuroinflammation, Rehabilitation

## Abstract

**Background:**

Ninjin’yoeito (NYT), a traditional Japanese Kampo medicine, improves the depression and anxiety in humans and animals, rendering it a novel therapeutic option for post-stroke depression (PSD). Furthermore, physical exercise is an important nonpharmacological therapy for major depressive disorder. The components of NYT or exercise exert antidepressant effects through the increased expression of neurotrophic factors and reduced neuroinflammation in the brain. However, the mechanisms underlying the antidepressant effects of NYT and exercise in PSD remain unclear. Therefore, we examined the effects of NYT and physical exercise in a rat model of PSD.

**Methods:**

Rats were divided into five groups: PSD, PSD with NYT, PSD with exercise (Ex), PSD with NYT and exercise (NYT + Ex), and control (sham). PSD was induced by the microinjection of endothelin-1 into the left medial prefrontal cortex and chronic unpredictable mild stress 3 days per week. A diet containing 3% NYT was administered to rats one day after stroke induction. Exercise was conducted using a motorized treadmill for three days per week, starting three days after the stroke. The therapeutic interventions lasted for four weeks. Serum corticosterone levels, depression-like behavior, and hippocampal pathophysiology, including the expression of brain-derived neurotrophic factor (BDNF), precursor BDNF (proBDNF), doublecortin (DCX), NeuN, glial cell activation, and tumor necrosis factor-α (TNF-α), were examined.

**Results:**

Serum corticosterone levels were lower in the treatment group than those in the PSD group. Notably, serum corticosterone levels were significantly lower in the NYT group than those in the PSD group. BDNF expression in the CA1 region was significantly higher in the Ex group than that in the PSD group. The NYT + Ex group showed a significantly higher hippocampal BDNF/proBDNF ratio than the other groups. DCX and NeuN expression levels were significantly higher in the NYT + Ex group than those in the NYT and PSD groups. Hippocampal glial cell activation and TNF-α expression increased in the PSD group and decreased in the intervention groups.

**Conclusions:**

NYT ameliorates serum corticosterone levels and hippocampal neuroinflammation in PSD. Additionally, this study suggested that NYT, together with exercise therapy, may improve neurogenesis, the BDNF/proBDNF ratio, and neuroinflammation in the hippocampus in PSD.

**Clinical trial number:**

Not applicable.

**Supplementary Information:**

The online version contains supplementary material available at 10.1186/s12906-025-04915-w.

## Background

Stroke survivors experience several stroke-related complications, including depression [1, 2], emotional and sleep disorders [3], fatigue [4], anxiety [5], apathy [6], and cognitive disorders [7]. These complications significantly affect patients’ long-term disabilities and emotional wellbeing [1]. Post-stroke depression (PSD) is a serious health problem, with nearly half of stroke patients suffering from long-term PSD [8, 9]. PSD is a common post-stroke mood disorder, and has a correlation with increased mortality and negatively correlates with functional recovery [1]. Even for PSD which is studied by most researchers among stroke-related complications, the mechanisms of PSD are not fully understand [1].

The pathophysiological mechanisms of PSD are multifactorial and complex and include hypothalamic–pituitary–adrenal (HPA) axis dysregulation, brain-derived neurotrophic factor (BDNF) deficiency, and excess inflammatory cytokines [8, 10]. The HPA axis is stimulated by proinflammatory cytokines to release glucocorticoids such as cortisol in patients with PSD [11]. Neurohormonal-inflammatory interactions exist between the HPA axis and the central nervous system (CNS), immune, and endocrine systems [10]. In addition, several studies have reported that hippocampal BDNF levels negatively correlate with acute and chronic stress and the incidence of PSD [12, 13]. BDNF is an essential neurotrophic factor that is widely observed in the mammalian brain and play an important role in development, synaptic plasticity, and neuronal survival. Animal studies have also demonstrated that rats with PSD exhibit decreased hippocampal BDNF levels and increased hippocampal neuronal apoptosis [12, 14, 15]. Recently, Liu S et al. [16] demonstrated that the RVG-modified exosomes engineered to overexpress BDNF alleviated depression-like symptoms in mice induced by intraperitoneal lipopolysaccharide injection. Furthermore, BDNF mRNA expression was decreased in the prefrontal cortex (PFC) of postpartum depression model mice, and injection of BDNF into medial PFC (mPFC) improved depression-like behaviors in the mice [17]. These studies suggest evidence of the therapeutic potential of BDNF in mood regulation. In contrast, precursor BDNF (proBDNF) with p75NTR may exert opposing effects, such as neuronal cell death and long-term synaptic depression through the activation of nuclear factor κB (NF κB), c-Jun kinase, and sphingomyelin hydrolysis [18, 19]. In addition, proBDNF negatively regulates hippocampal dendric complexity and spine density through p75NTR [20]. Therefore, increased hippocampal proBDNF levels may play an important role in the pathogenesis of PSD. Relative levels of hippocampal BDNF and proBDNF may be associated with the development of PSD [12]. In clinical study, major depressive patients exhibit higher serum levels of proBDNF and p75NTR compared to healthy controls, while they exhibit lower levels of BDNF and tropomyosin receptor kinase B (TrkB) [21]. Therefore, the BDNF and proBDNF may be potential therapeutic target. In addition, brain inflammation is a risk factor for PSD [22]. Microglia and astrocytes mediate innate immune responses in the CNS. Chronic stress promotes the hippocampal microglial activation in mice [23]. Microglial activation is a major trigger of depression in both humans and animal models [24–26], suggesting that the hippocampal region may exhibit increased inflammation during PSD. Taken together, PSD is caused by a variety of interrelated neurochemical and anatomical alterations in the brain. Notably, inflammatory conditions are associated with the HPA axis disfunction and brain BDNF levels. Furthermore, reduction BDNF reduces neurogenesis and synaptic plasticity in the hippocampus.

Currently, medication is the preferred choice for the management of PSD. Antidepressants, such as fluoxetine, selective serotonin reuptake inhibitors (SSRI), and sertraline, are effective in alleviating depressive symptoms and improving sleep quality; however, they have serious side effects [10]. Several studies have demonstrated the effectiveness of complementary and alternative medicines such as herbal Kampo medicines in treating patients with anxiety and depression [27, 28]. Herbal Kampo medicines have fewer adverse effects than antidepressants.

Ninjin’yoeito (NYT) is a traditional Japanese Kampo medicine comprising of 12 herbs: *Rehmannia* root, Japanese *angelica* root, *Atractylodes* rhizome, *Poria* sclerotium, ginseng, cinnamon bark, *Polygada* root, peony root, *Citrus unshiu* peel, *Astragalus* root, *Glycyrrhiza*, and *Schisandra* fruit. NYT has multifaceted beneficial effects, such as improving depression, apathy, fatigue, anemia, night sweats, and anorexia, and promoting recovery from several diseases [29]. NYT is used for individuals with deteriorated psychiatric or physical conditions, particularly older patients in Japan [30]. The patients with PSD have substantial overlapped in prevalence of some complications, such as symptoms of anxiety, apathy, and fatigue [31]. Therefore, NYT may have beneficial effects not only on depression but also on overlapped complications. In recent clinical studies, NYT treatment has been shown to alleviate Alzheimer’s disease-related depression and improve cognitive outcomes [27]. NYT improved postpartum fatigue and anemia, and prevented the development of postpartum depression [28]. In addition, NYT treatment in older patients is safe and significantly improves fatigue, malaise, and anorexia [30]. In preclinical studies, NYT attenuates behavioral abnormalities through hippocampal neurogenesis in a mouse depression model [32]. NYT administration improved hippocampal BDNF levels, which ameliorated depression-like behavior and anxiety in aged mice with chronic obstructive pulmonary disease-induced anxiety and depression [33]. NYT improves diminished motivation in apathy model mice [34]. NYT reduced fatigue-like conditions by alleviating inflammation in the brains of aging mice [35]. In addition, each herbal ingredient of NYT has various effects, including anti-inflammatory, antioxidant, and neuroprotective effects that has been demonstrated in animal models of neurological diseases [29]. For instance, Ginsenoside Rd, the most active component of ginseng, exert antidepressant-like effects through the BDNF/ tropomyosin receptor kinase B (TrkB) signaling pathway in chronic social defeat stress model mice [36]. The extracts of *Polygala tenuifolia* and *Panax ginseng* promote hippocampal neurogenesis through the BDNF/TrkB signaling pathway [37]. NYT increased nerve growth factor (NGF) levels in cultured rat astrocytes, and *Polygala* root or *Panax ginseng* extracts significantly increased NGF levels compared with other NYT ingredients [38]. Schizandrin, NYT component, is responsible for its anxiolytic effects in neuropeptide Y-knockout zebrafish that exhibit severe anxiety responses [39]. Several in vitro [40, 41] and in vivo [42] studies have reported that herbal ingredient of NYT have neurotrophic, neurogenesis, and neuroprotective effects. In addition, several components of NYT, such as paeoniflorin, glycyrrhetinic acid, and schizandrin, may be transported across the blood brain barrier into the brain after oral administration. [43–45]. However, the effects and mechanisms of action of NYT in PSD remain unclear. NYT and NYT ingredients may be improving the depressive symptoms and pathophysiological manifestations of PSD, rendering it a novel therapeutic option for PSD.

Pharmacological and non-pharmacological therapies are available for patients with PSD [46]. Exercise-based interventions and aromatherapy have been shown to be useful non-pharmacological therapies for improving patients with PSD [47, 48]. In preclinical studies using a rat stroke model, physical exercise improved functional recovery by reducing neuroinflammation and increasing BDNF expression around the lesions [49]. Exercise may be reduced the number of microglia and inhibiting microglia activation and hippocampal neuroinflammation in a depression model rat [50]. Aerobic exercise may ameliorate depression and hippocampal neurogenesis and increase hippocampal BDNF expression in a rat model of PSD [12]. In addition, NYT, in combination with physical exercise, improved sensorimotor function by stimulating BDNF/ TrkB and NGF/ TrkA, and by activating the Akt pathway in stroke models [51]. These studies suggest that the combination of NYT and physical exercise provide additive or synergistic effects in improving PSD symptoms and its pathophysiological alterations. However, the effects of complementary NYT and rehabilitative exercises on PSD remain unclear.

There are several animal models of PSD, including middle cerebral artery occlusion combined with chronic unpredictable mild stress (CUMS) or mPFC ischemia. Recent studies have reported a positive relationship between PSD and mPFC damage following endothelin-1 (ET-1) injection [52–54]. Various brain areas are involved in projections from the ventral tegmental area to the nucleus accumbens and mPFC to induce depression-like behavior [55]. The auditory cortex, amygdaloid nucleus, anterior cingulate cortex, hippocampus, hypothalamus, and bed nucleus of the stria terminalis are associated with specific aspects of fear- and anxiety-related behaviors [55]. Unilateral ET-1 lesions in the mPFC have been demonstrated to cause persistent depression-like phenotypes in mice [52]. However, Happ et al. [53] reported that unilateral damage to the mPFC in rats did not induce a persistent depression-like phenotype. Therefore, this study used unilateral ET-1 lesions in the mPFC, in combination with chronic CUMS, as a model of PSD.

This study aimed to determine the effects of NYT administration and physical exercise on serum corticosterone levels, depression-like behavior, and hippocampal pathophysiology in a rat model of PSD.

## Methods

### Animals

Forty-seven adult male Sprague–Dawley rats, aged 8–9 weeks and weighing 327.8 ± 23.3 g (mean ± SD) at the time of surgery, were purchased from the Japan SLC, Inc. animal suppliers (Hamamatsu, Japan). Animals were housed in cages with a 12-h light/dark cycle and kept in temperature-controlled conditions (23.0 ± 1.0℃), with food and water available ad libitum. This study was approved by the Animal Care Committee of the Kagoshima University (No. M22002), and performed in compliance with the ethical guidelines of the Institute of Laboratory Animal Sciences of Kagoshima University.

### Experimental protocol

**Fig. 1:**
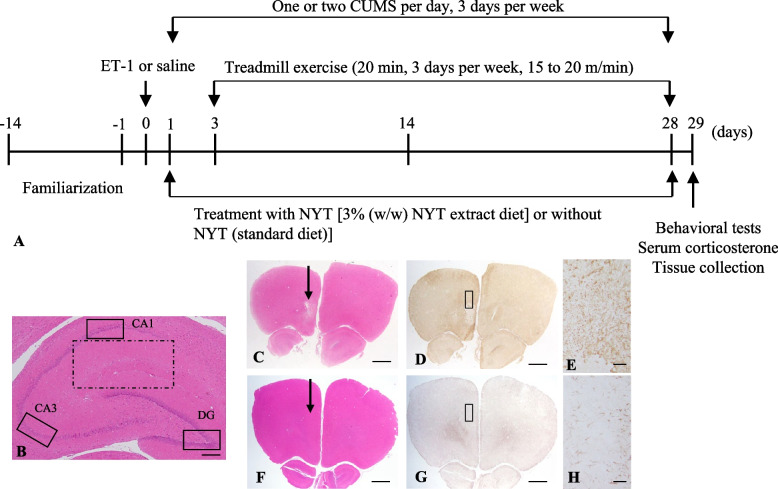
Experimental protocol. **A** Timeline of NYT administration (1 to 28 days after stroke) and treadmill exercise (3 to 28 days after stroke). **B** Neurons in the CA1, CA3, dentate gyrus (DG) regions (line squares), and grail activations (dash square) in both hippocampi were assessed. **C**, **D**, **F**, **G** Focal lesion (arrow) following ET-1 injection and sham into the left mPFC shown using HE (C, F) and GFAP immunohistochemistry (D, G,). **E**, **H** High magnification of D and G (rectangle) Scale bar = 1 mm (B), 250 µm (C, D, F, G), 50 µm (E, H)

The experimental procedure is illustrated in Fig. 1A. All animals were acclimatized for 14 days before the experimental began. After acclimation, the animals were assigned to five groups: stroke with CUMS (PSD group, *n* = 10), PSD with NYT treatment (NYT group, *n* = 10), PSD with physical exercise (Ex group, *n* = 10), and PSD with NYT and physical exercise (NYT + Ex group, *n* = 9). Sham animals were used as controls (sham, *n* = 8). The NYT extract was mixed with a standard diet (Oriental Yeast Co., Tokyo, Japan) at a concentration of 3% (w/w) and fed to the rats from 1 to 28 days after stroke. Rats in the PSD, Ex, and sham groups were fed a standard diet without NYT from 1 to 28 days after stroke. Behavioral tests, serum corticosterone levels, and brain tissue analysis were performed 29 days after stroke. The rats were fed 40 g of the diet per day. Food intake was measured, and the mean weekly food intake was calculated. Body weight was measured periodically during the experiment.

### Stroke induction

Rats were anesthetized with 2–3% isoflurane using small animal inhalation anesthesia machine (MK-A110D, Muromachi Kikai Co., LTD, Japan) and placed in a stereotactic head holder SR-5N (Narishige, Tokyo, Japan). The dorsal surface of the skull was exposed by a midline incision, and a small burr hole (2 mm in diameter) was drilled in the left mPFC (anterior: 3.0 mm, medial: 1.0 mm, depth: 4.0 mm relative to bregma). Then, a Hamilton syringe (75N 87900) was put into the mPFC, and 1 µl of ET-1 (400 pmol/µl, 4189-s, Peptide Institute, Inc, USA) or 1 µl of physiological saline was injected over 1 min, and then left for 4 min to prevent solution leakage. Physiological saline was used as a sham model.

### Chronic unpredictable mild stress (CUMS)

After stroke induction, the animals were individually housed and subjected to the following CUMS: tail clipping for 10 min, tail suspension for 20 h, water withheld for 24 h, vibration of the cage for 5 min at a frequency of once per second, tilting at 20° for 24 h, and placement in wet bedding for 24 h. The animals underwent one or two CUMS stimulation sessions per day, 3 days per week, from 1 to 28 days after stroke. The CUMS was administered 12 times during the study period. The CUMS and exercise were not performed on the same day. The sham group was housed with two animals per cage and kept in clean cages without interference.

### Ninjin’yoeito (NYT)

NYT (lot no. 39228490) was obtained from Tsumura & Co. (Tokyo, Japan). To prepare 6 g of NYT extract powder, a mixture of 12 dried natural components was used to manufacture a spray dried powder from the hot water extract (yield 19%) (Table 1). Plant materials were verified by identifying marker compounds (glycyrrhizic acid, paeoniflorin, and hesperidin), and plant specimens were identified based on their external morphology according to the Japanese Pharmacopeia and company standards. The extract quality was standardized based on good manufacturing practices as defined by the Ministry of Health, Labor, and Welfare of Japan. The detailed plant source and extensive profiling of NYT ingredients using three-dimensional HPLC (Tsumura & Co) are shown in Additional File 1. Plant names were confirmed using the MPNS (http://mpns.kew.org) or “Real-World Flora Online” (www.worldfloraonline.org).

**Table 1:** The galenical components of Ninjin’ yoeito (NYT)

Latin name of crude drug	Original plant source and medical parts of crude drug	Amount (g)
Rehmanniae Radix	The roots of *Rehmannia glutinosa *Libosch. var.* purpurea *Makino or *Rehmannia glutinosa* Liboschitz	4.0
Angelicae Acutilobae Radix	The roots of *Angelica acutiloba* Kitagawa or *Angelica acutiloba* Kitagawa var. *sugiyama* Hikino	4.0
Atractylodis Rhizoma	The rhizome of *Atractylodes japonica* Koidzumi ex Kitamura or *Atractylodes macrocephala* Koizumi (*Astractylodes ovata* De Candolle)	4.0
Poria	The sclerotium of *Wolfiporia cocos* Ryvarden et Gilbertson (*Poria cocos* Wolf)	4.0
Ginseng Radix	The roots of *Panax ginseng* C. A. Meyer (*Panax schinseng* Nees)	3.0
Cinnamomi Cortex	The bark of *Cinnamomun cassia* Blume	2.5
Polygalae Radix	The roots of *Polygala tenuifolia* Willdenow	2.0
Paeoniae Radix	The roots of *Paeonia lactiflora* Pallas	2.0
Gitri Unshiu Pericarpium	The pericarp of *Citrus unshiu* Markowicz or *Citrus reticulata* Blanco	2.0
Astragali Radix	The roots of *Astragalus membranaceus* Bunge or *Astragalus mongholicus*	1.5
Glycyrrhizae Radix	The roots and stolons of *Glycyrrhiza uralensis* Fischer or *Glycyrrhiza glabra *Linne.	1.0
Schisandrae Fructus	The fruits of *Schisandra chinensis* Baillon	1.0

The weights indicate the amount of each herbal medicine used to produce 6 g of dry NYT extract

### Physical exercise

Three days after stroke induction, the Ex groups were trained using a treadmill (MK-680, MUROMACHI KIKAI Co., Ltd., Japan) for 20 min/day, 3 days/week for 4 weeks **(**Fig. 1A**)**. Exercise intervention was performed 12 times during the experimental period. The Animals exercised at a speed of 15–20 m/min on the first three occasions, and the running speed was increased to 20 m/min on the remaining exercise occasions, which were performed during the day.

### Serum corticosterone

Serum corticosterone levels were measured to evaluate stress-associated hormonal imbalance in each group four weeks after stroke. The blood sample with the coagulation accelerator was left at room temperature for 30–120 min and centrifuged at 3,000 g × for 10 min. The supernatant was transferred to a fresh tube and stored at −80℃ until analysis. Serum corticosterone concentrations were estimated using an enzyme-linked immunosorbent assay (ELISA) by Yanaihara Institute, Inc. (Fujinomiya, Japan). Blood samples were obtained from all animals between 10 am and 1 pm.

### Neurological deficits and behavioral tests

Neurological deficits were evaluated using a 5-point neurological grading score (0–4), as described previously [56]. Depression-like behaviors were assessed using the open field, Y-maze, and sucrose preference tests before and four weeks after stroke.

The open field test was used to assess anxiety in rats, as previously described [57]. Briefly, the animals were placed in the center of the open field and spontaneous activity was recorded for 10 min using a video camera (Logicool HD Pro Webcam C920r). The locomotion distance and time spent in the center zone (15 × 20 cm) were analyzed using SMART (version 3.0; Panlab, Barcelona, Spain).

The Y-maze test was used to assess short-term memory. The apparatus consisted of three identical arms (51.5 × 10 × 25 cm) symmetrically separated at 120° (YM-03R; MUROMACHI KIKAI Co., Ltd., Japan). Animals were put on the end of the arm and were free to explore all three arms; the behavior was recorded using a video camera. The number of arms visited and alteration of arm visits were recorded visually for 6 min using a SMART (version 3.0; Panlab, Barcelona, Spain). Arm entry was defined as entry of a rat’s body into the arm. Alteration was defined as entry into all three arms on consecutive occasions. The percentage of alteration was evaluated using the following formula: actual alteration/visual alteration (defined as the total number of arm entries 2) × 100.

The sucrose preference test was used to evaluate anhedonia (lack of pleasure) and depressive symptoms. Two bottles containing different liquids (water and a 1% sucrose solution) were placed in the cage simultaneously. After 20 h of water deprivation, rats were allowed free access to the bottles for 24 h. The consumption of each liquid was recorded, and sucrose preference was evaluated using the following formula: sucrose intake (ml)/total liquid intake (ml) × 100.

### Tissue preparation

The animals were anesthetized via intraperitoneal injection of 5% sodium pentobarbital and then transcardially perfused with 0.9% saline before decapitation. The whole brain was sliced into seven 2-mm-thick coronal sections. Brain sections were fixed in 4% paraformaldehyde in 0.1 M phosphate buffer (pH 7.4) at 4 ℃ overnight. The fifth coronal section of seven consecutive TTC sections from the cranial to the caudate region, including the hippocampus, was used for histological and immunohistochemical analyses. The sixth and seventh coronal sections including the remaining left hippocampus, were used for western blot analysis.

### Histology and immunohistochemistry

Coronal brain sections were stained with hematoxylin and eosin (HE) and the following antibodies: rabbit anti-BDNF (Bioss, Inc, bs-4989R), rabbit anti-ionized calcium-binding adapter molecule 1 (Iba-1, a marker of microglia) antibody (Wako, 019–19741), rabbit anti-glial fibrillary acidic protein (GFAP: a marker of activated astrocytes) antibody (Gene Tex, Inc., GTX108711), and rabbit anti-doublecortin (DCX: marker of neurogenesis) antibody (Abcam plc, ab18723) in accordance with the manufacturers’ instructions. The sections were incubated with antibodies at the following dilution ratios at 4 ℃ overnight: rabbit anti-BDNF antibody (1:500), rabbit anti-Iba-1 antibody (1:1000), rabbit anti-GFAP antibody (1:2000), and rabbit anti-DCX antibody (1:1000). After washing with phosphate-buffered saline (PBS), the sections were reacted with goat anti-rabbit IgG conjugated to a peroxidase-labeled dextran polymer (EnVision, Dako, CA, USA) for 60 min and immunoreactivity was detected using diaminobenzidine staining.

The BDNF-immunostained sections in the CA1, CA3, and dentate gyrus (DG) of the hippocampus were imaged at 20 × magnification, and the DCX-immunostained sections in the DG of both hippocampi were imaged at 20 × magnification (Fig. 1B, solid square). The hippocampus, including the stratum radiatum and lacunosum in the Iba-1- and GFAP-immunostained sections, was imaged at 10 × magnification (Fig. 1B, dashed square). The ratios of BDNF-, DCX-, Iba-1-, and GFAP-positive areas were quantitatively estimated using ImageJ software (1.54 h, NIH, USA), and the average ratio of immunopositive areas in both hippocampi was used for statistical analysis. Quantitative analysis was performed by three individuals who were blinded to the group allocation.

### Western blot analysis

Western blotting was conducted to evaluate protein levels in the left hippocampus (*n* = 3–5 in each group), as described previously [51]. The membrane was reacted with the following primary antibodies: rabbit anti-BDNF (1:1000, bs-4989R; Bioss, Inc.), mouse anti-proBDNF (1:1000, Santa Cruz Biotechnology, INC., sc-65514), rabbit anti-Iba-1 (1: 1000, Wako, 016–20001), rabbit anti-GFAP (1:1000, Cosmo Bio Co, Ltd, SML-RO1003), rabbit anti-NeuN (1:1000, Cell Signaling Technology, #24,307), rabbit anti-tumor necrosis factor-α (TNF-α, a marker of proinflammatory cytokine, 1:500, Abcam plc, ab183218) and mouse anti-α-tubulin (1:5000, 66,031–1-Ig; Proteintech, USA) at 4 °C overnight and then with a secondary horseradish peroxidase-labeled antibody for 1 h at room temperature. Protein bands were detected by chemiluminescence (WSE-6100 LuminoGraph I; ATTO), visualized using an EzWestlumi plus detection system (ATTO), and semi-quantitatively measured using ImageJ software.

### Statistics

The ratios of weight gain, total locomotion distance, alternation triplet, and BDNF expression in the CA3, Iba-1, and GFAP were analyzed by one-way analysis of variance (ANOVA), followed by Tukey’s post hoc multiple comparisons. Feed intake, serum corticosterone level, time spent in the central areas, sucrose performance, and expression of BNDF in CA1, DG, TNF-α, and DCX were analyzed using the Kruskal–Wallis test, followed by Dunn’s post hoc test. Statistical significance was set at *p* < 0.05. Statistical analyses were performed using parametric or nonparametric tests after the Shapiro–Wilk test. Outlier removal was performed using the ROUT method in the GraphPad Prism software. GraphPad Prism software version 10.1.2 for Windows (San Diego, California, USA) was used for data analyses. All data are presented as mean ± standard error (SEM).

## Results

### Effect of ET-1- injection on the mPFC

ET-1 was microinjected into the left mPFC, and a focal lesion was observed in the mPFC four weeks after the injection (Fig. 1C, D). These lesions differed from those observed in the sham control group (Fig. 1F, G). GFAP immunostaining was performed to investigate glial activation surrounding the lesion. GFAP immunoreactivities of the ET-1 injected rats were significantly increased in the surrounding lesions compared to those of the sham rats and the contralateral sides (Fig. 1E, H).

The neurological score was almost 0 (no apparent deficits) 1 day after the stroke. Only two animals (5%) exhibited score 1 (right forelimb flexion) on 1 day after stroke; however, these animals had a score of 0 within 2 weeks after stroke, suggesting that the unilateral ET-1-induced mPFC stroke model does not result in sensorimotor deficits.

### Effect of NYT and exercise on food intake and body weight

**Fig. 2:**
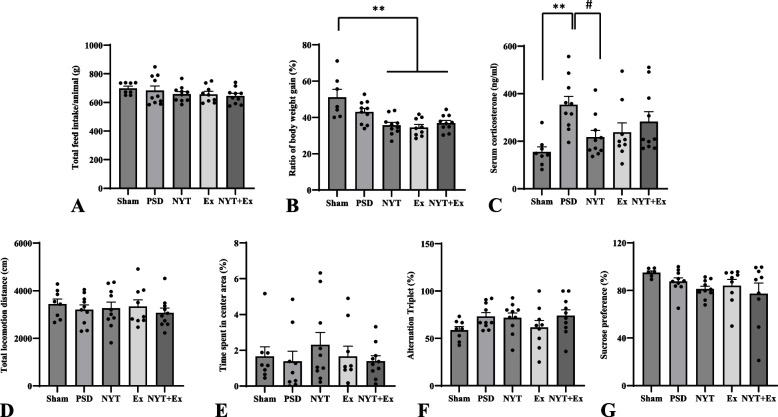
Effect of NYT and exercise on feed intake, ratio of body weight gain, serum corticosterone level, and depression-like behavior in the PSD rats. **A** Feed intake. **B** Ratio of body weight gain. **C** Serum corticosterone level in the PSD group was significantly increased compared to that in the sham group. **D** Total locomotion distance. **E** The ratio center zone entry times. **F** Y-maze test (percentage of alterations). **G** Sucrose preference test. Mean ± SE. ** *p* < 0.01 (ANOVA between 5 groups). # *p* < 0.05 (ANOVA between PSD animals). (*n* = 9–10 in each group)

All rats were fed a diet with or without NYT 1 day after ET-1 or saline injections. There was no significant difference in feed intake per week between the groups during the experiment (Additional File 2). Feed intake varied among the individuals in each group. The mean daily food intake per day for all groups during the experiment was 23.9 ± 3.3 g (mean ± SD). Some PSD rats had higher total feed intake than the sham group, particularly those in the PSD group (Fig. 2A). However, no significant differences were observed in the total feed intake during the experimental period, suggesting that the overall drug dose was the same in each group (Fig. 2A).

Body weight increased throughout the experiment (Additional File 2). No significant differences were observed between the groups at four weeks post-stroke. However, the ratio of body weight gain from before to the end of the experiment was reduced in PSD animals compared to the sham group (Fig. 2B). Notably, the ratio of body weight gain in the therapeutic groups was significantly lower than that in the sham group (*p* < 0.01).

### Effect of NYT and exercise on serum corticosterone

High glucocorticoid levels are also associated with depression [10]. Therefore, we investigated whether serum corticosterone concentrations increased or decreased in PSD after therapeutic interventions. Serum corticosterone levels in the PSD rats were higher than those in the sham group (Fig. 2C). Notably, the PSD group had significantly higher serum corticosterone levels than the sham group (*p* < 0.01). The corticosterone levels in the therapeutic groups were lower than those in the PSD group (Fig. 2C). Notably, the serum corticosterone levels in the NYT group were significantly lower than those in the PSD group (*p* < 0.05).

### Effect of NYT and exercise on depression-like behavior

To evaluate the efficacy of NYT and exercise on depression-like behavior, we performed three tests (the open field test, Y-maze test, and sucrose preference test). The NYT group indicated an increase in the ratio of time for which the rats entered the central zone. However, there was no significant difference in the distance traveled or ratio of time spent in the central zone between the groups (Fig. 2D, E). In addition, no significant differences were observed in the Y-maze test (percentage of alterations) or the sucrose preference test between the groups (Fig. 2F, G).

### Effect of NYT and exercise on the hippocampal BDNF/proBDNF

**Fig. 3:**
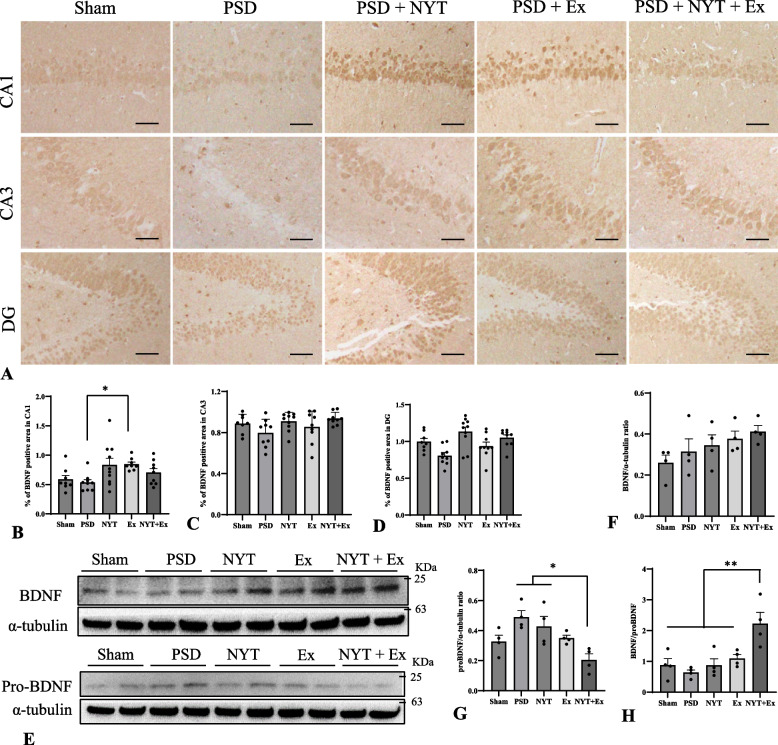
Effect of NYT and exercise on the hippocampal BDNF/proBDNF expressions. **A-D** immunohistochemical analyses of BDNF positive cells in the hippocampal CA1 (B), CA3 (C), and dentate gyrus (DG, D). **E–H** Protein levels of BDNF (F) and proBDNF (G), and BDNF/proBDNF ratio (H) in the hippocampus. Scale bar = 50 µm (A). Mean ± SE. * *p* < 0.05, ** *p* < 0.01. (*n* = 4–10 in each group)

We determined BDNF and pro-BDNF expression in the hippocampus of PSD rats using immunohistochemistry and western blotting (Fig. 3). The mean ratio of the BDNF-immunoreactive areas in each hippocampal region was lower in the PSD group than in the sham group. In contrast, the hippocampal BDNF-immunoreactive areas in the therapeutic groups were larger than those in the PSD group (Fig. 3A-D). Notably, BDNF expression in the CA1 region was significantly higher in the Ex group than that in the PSD group (Fig. 3A; *p* < 0.05). Additionally, BDNF expression in the DG was higher in the therapeutic groups than in the PSD group (Fig. 3D). Hippocampal BDNF protein levels were higher in the therapeutic groups than in the PSD group (Fig. 3E). However, no significant differences were observed between groups (Fig. 3F). Protein levels of proBDNF were higher in the PSD group than in the sham and therapeutic groups (Fig. 3E, G). The NYT + Ex group showed a significant decrease in the protein level of hippocampal proBDNF compared to the PSD group (Fig. 3G; *p* < 0.05). To examine the effects of the balance between the hippocampal BDNF and proBDNF levels, we assessed the relative levels of BDNF and proBDNF. The PSD group had a decreased mean BDNF/proBDNF ratio compared to the sham group (Fig. 3H). The NYT (0.87 ± 0.21; mean ± SE) and Ex (1.09 ± 0.13) groups showed an increased BDNF/proBDNF ratio compared to the PSD (0.63 ± 0.08) group. Notably, the NYT + Ex (2.22 ± 0.37) group had a significantly increased BDNF/proBDNF ratio compared to the other groups (Fig. 3H; *p* < 0.01).

### Effect of NYT and exercise on hippocampal neurogenesis

**Fig. 4:**
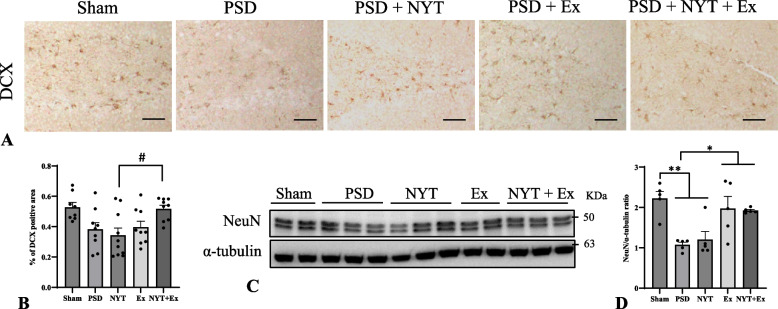
Effect of NYT and exercise on hippocampal neurogenesis. **A**, **B** immunohistochemical analyses of DCX-positive cells in the hippocampal DG. **C**, **D** Protein levels of NueN in the hippocampus. Scale bar = 50 µm (A). Mean ± SE. * *p* < 0.05, ** *p* < 0.01 (ANOVA between 5 groups). # *p* < 0.05 (ANOVA between PSD animals). (*n* = 4–10 in each group)

In addition, we investigated the expression of DCX in the DG and protein levels of NeuN in the hippocampus (Fig. 4A). The ratio of the DCX-immunoreactive areas was significantly higher in the NYT + Ex (0.52 ± 0.02%) group than in the NYT (0.34 ± 0.05%) group (Fig. 4B; *p* < 0.05), suggesting a possible synergistic effects of NYT and physical exercise in modulating neurogenesis. NeuN protein levels were significantly lower in the PSD (1.07 ± 0.06%) and NYT (1.20 ± 0.20%) groups than in the sham (2.22 ± 0.17%) group (Fig. 4C, D; *p* < 0.01). However, NeuN expression was significantly higher in the Ex (1.97 ± 0.30%) and NYT + Ex (1.92 ± 0.03%) groups than in the PSD group (Fig. 4C, D; *p* < 0.05).

### Effect of NYT and exercise on hippocampal neuroinflammation

**Fig. 5:**
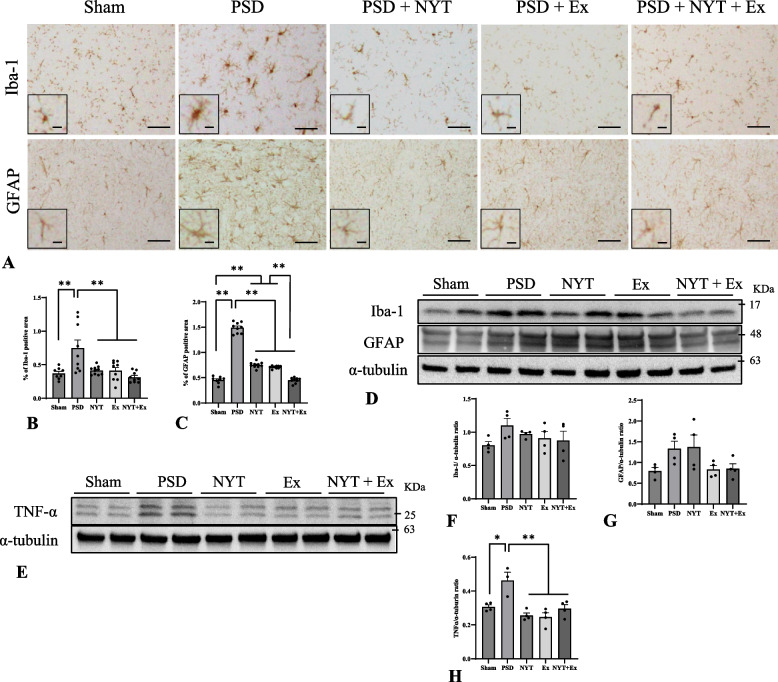
Effect of NYT and exercise on hippocampal glial activation and neuroinflammation. **A-C** immunohistochemical analyses of Iba-1- and GFAP-positive cells in the hippocampus. **D**, **F**, **G** Protein level of Iba-1 and GFAP in the hippocampus. **E**, **H** Protein level of TNF-α in the hippocampus. The protein level of hippocampal TNF-α was increased in the PSD group. The expression of TNF-α in the NYT, Ex, and NYT + Ex groups were significantly lower than that in the PSD group. Scale bar = 50 µm (A), 10 µm (high magnification in A). Mean ± SE. * *p* < 0.05, ** *p* < 0.01. (*n* = 3–10 in each group)

Finally, we examined the expression of Iba-1-positive microglia and GFAP-positive astrocytes in the hippocampus. Immunohistochemistry revealed that the number of activated microglia and astrocytes increased in the hippocampi of the PSD group compared with those in the sham and therapeutic groups (Fig. 5A). The ratios of the Iba-1- and GFAP-immunoreactive areas were significantly higher in the PSD group than in the sham and therapeutic groups (*p* < 0.01; Fig. 5B, C). In contrast, the ratios of the Iba-1-and GFAP-immunoreactive areas were significantly lower in the therapeutic groups than in the PSD group (*p* < 0.01; Fig. 5B, C), suggesting a possible synergistic effects of NYT and exercise in modulating the PSD-related hippocampal neuroinflammation. The protein levels of hippocampal Iba-1 showed a trend similar to that of immunohistochemical analyses (Fig. 5D, F). Hippocampal GFAP protein levels were lower in the Ex and NYT + Ex groups than in the PSD group (Fig. 5D, G). In addition, the protein levels of hippocampal TNF-α were increased in the PSD group. TNF-α expression was lower in the therapeutic groups than that in the PSD group (Fig. 5E, H).

## Discussion

This study demonstrated the efficacy and possible mechanisms of NYT administration in the pathophysiological manifestations of PSD. NYT significantly decreased serum corticosterone levels in rats with PSD. The HPA axis is a key factor linking depression and stress [10]. Stress increases the activity and expression of hypothalamic corticotropin-releasing hormone (CRH) neurons, resulting in an increased release of CRH, which in turn stimulates the increased secretion of downstream adrenal cortical hormone (ACTH) and corticosterone, eventually leading to hyperactivity of the HPA axis [11, 58]. Therefore, this study suggests that NYT administration may ameliorate the impaired HPA axis function. In addition, this study suggests that NYT administration attenuates hippocampal necroinflammation, which may be related to the amelioration of depression and stress as well as impaired HPA axis function. Notably, our findings showed that NYT combined with physical exercise increased the hippocampal BDNF/proBDNF ratio and neurogenesis, and decreased hippocampal neuroinflammation. BDNF deficiency and increased hippocampal proBDNF levels contribute to the pathophysiological manifestations of PSD [10, 20, 59]. Hippocampal neuroinflammation plays an essential role in the pathogenesis of depression [60]. Running mediates the inhibition of hippocampal neuroinflammation in a stress-induced depression model [50]. Our results showed that the combined therapy of NYT and exercise did not interfere with its beneficial effects on the hippocampus. Therefore, this study suggests that NYT together with physical exercise may be effective in improving the pathophysiological hippocampal environment in PSD by decreasing neurotrophic factors, neuronal loss, and neuroinflammation. Both treatments, NYT and physical exercise, may interact at the molecular level and enhance each other’s effects.

Vahid-Ansari et al. [52] reported that impaired mPFC function caused by unilateral ET-1 injection in mice induces depression- and anxiety-like behaviors. However, Happ et al. [53] reported that impaired mPFC function caused by a unilateral ET-1 injection in rats resulted in fewer depression- and anxiety-like behaviors. In addition, rats with ischemic lesions in both the mPFC and nucleus accumbens displayed fewer depression-like behaviors. Our results showed that ischemic lesions in the unilateral mPFC and stimulation with CUMS resulted in fewer depression-like behaviors. Several studies have used middle cerebral artery occlusion/reperfusion (MCAO) and CUMS as PSD models in rats [12, 14, 20]. CUMS has also been used as a model for stress-induced depression in rats [61]. Taken together, unilateral mPFC lesions or MCAO alone may have no apparent short- or long-term effects on locomotion, cognition, or depression-like behaviors in rats, in contrast to what has been demonstrated in mice. Further studies are required to examine the effects of NYT and/or exercise on depression-like behaviors using mouse models of depression. However, our model showed that the serum corticosterone levels increased in response to CUMS. In addition, the mean ratio of time spent in the center area in the open-field test was reduced in PSD rats. These findings suggest that impaired HPA axis function may lead to depression and anxiety.

Hippocampal BDNF downregulation is involved in the pathophysiology of depression [12, 59]. In a stress model, BDNF mRNA levels were reduced in the DG and hippocampus of rats [62]. Antidepressant treatment increase BDNF synthesis [13], and NYT components exert antidepressant effects by enhance hippocampal BDNF levels [37]. Our results show that NYT and/or exercise increased the number of BDNF-positive cells in the DG, suggesting that increased BDNF levels in the DG mediate neuronal survival and neurogenesis. However, NYT and exercise alone did not increase in the expression of DCX and NeuN. Previous studies have shown that physical exercise increases the upregulation of brain neurotrophic factors such as BNDF and NGF after stroke [63, 64]. Therefore, further studies are required to elucidate the beneficial effects of NYT components and exercise on PSD. Our previous study demonstrated that NYT, in combination with exercise, exerts neuroprotective effects by enhancing BDNF and NGF levels after stroke in rats [51]. Therefore, NYT combined with exercise, but not alone, may improve hippocampal BDNF levels, neurogenesis, and neuronal loss in patients with PSD.

Additionally, proBDNF primarily binds to the p75NTR, inducing neuronal apoptosis and depression [20]. Considering the opposing functions of BDNF and proBDNF in hippocampal neurogenesis, relative levels of hippocampal BDNF/proBDNF may be associated with PSD [12]. Our results showed that the hippocampal BDNF/pro-BDNF ratio decreased in the PSD group. In contrast, the hippocampal BDNF/proBDNF ratio increased in the therapeutic groups. Notably, NYT combined with exercise significantly increased the hippocampal BDNF/proBDNF ratio. The BDNF/proBDNF ratio is a biomarker involved in the promotion of hippocampal neuroplasticity [65]. Therefore, the balance in the hippocampal BDNF/proBDNF ratio may play a pivotal role in the pathophysiology of PSD.

Neuroinflammation is caused by numerous factors such as stress and infection. Glial cells, including microglia and astrocytes, are the primary mediators of immune responses in the CNS, and are thought to play a functional role in the pathophysiology of depression [66]. This study also showed that glial cells were activated in the hippocampi of the PSD group. In animal studies, inducible nitric oxide synthase (iNOS) and interleukin (IL)−1β released by microglia promote neuroinflammation in depression, leading to neurotoxicity and pathological alterations [67]. NYT prevented the onset of depression-like behaviors by decreasing hippocampal iNOS levels in a mouse model of Alzheimer’s disease [68]. Our immunohistochemical analyses showed that administration of NYT reduced hippocampal microglial and astrocyte activation. In addition, the protein levels of TNF-α, inflammatory cytokine, was decreased in the hippocampus by the therapies. These results suggest that NYT and/or exercise attenuate hippocampal neuroinflammation in PSD. A recent study reported that the antidepressant effect of running may be mediated by the inhibition of microglial activation and neuroinflammation in the hippocampus [50]. Therefore, NYT, together with exercise therapy, may attenuated hippocampal neuroinflammation in patients with PSD.

Our results showed that exercise slightly increased mean serum corticosterone levels. Notably, NYT, in combination with physical exercise, increased corticosterone levels. These results suggest that treadmill exercise induces mild stress in animals. Therefore, exercise regimens should consider the intensity, frequency, and duration of PSD. A moderate physical exercise regimen was administered in this study. Moderate physical exercise improves age-related cognitive function in mice by increasing hippocampal BDNF levels and suppressing neuronal loss, oxidative stress, and neuroinflammation [57]. Further studies are required to explore the effects of exercise regimens on hippocampal pathology in PSD.

The body weights of patients with depression often decrease because of anorexia or sleep disorders. However, the ratio of body weight gain was higher in the PSD group than in the therapeutic group. Some rats in the PSD group may have been overeaten because of stress after stroke. Happ et al. [53] reported that unilateral ET-1 damage alone or in combination with nucleus accumbens damage in a rat model of depression did not decrease body weight until six weeks. Similarly, we did not observe a decrease in body weight. NYT is prescribed to ameliorate several symptoms, such as anorexia and fatigue [29], which are commonly observed in patients with depression. Consequently, further investigations are needed to explore the anti-anorexic effects of NYT and exercise using other PSD models.

This study has some limitations. Immunohistochemical analyses were performed on both hippocampi because they communicate via the corpus callosum. Therefore, it may be necessary to compare differences in the hippocampi on both sides. Second, this study investigated neurotrophic factors, neurogenesis, and neuroinflammation in the hippocampi of PSD. Further studies are required to clarify the complex pathological mechanisms underlying PSD, including proinflammatory cytokines and microglial polarization. Third, NYT was mixed with the standard diet in the study. To avoid stressing the animals, we did not restrict food intake, nor did we use intraperitoneal injections or forced oral gavage. Further studies are need to examine the effects of different dose of NYT, long-term effects of NYT, and the effects of other forms exercise. Fourth, we aimed to minimize the number of animals used in this study. Therefore, some experiments were performed with relatively small sample sizes, which may have affected the reliability of the findings. Fifth, women are generally more susceptible to depression than men. Therefore, further experiments are required to examine sex differences in the responses to depression treatment. Sixth, no depression-like behavior was clearly observed in the rat PSD model. Therefore, behavioral tests did not show any statistically significant antidepressant effects. Further studies are required to clarify the validity of NYT and physical exercise as effective antidepressants against depression-like behavior in PSD. Despite these limitations, the results of the present study suggest that NYT attenuates serum corticosterone levels in PSD. In addition, NYT and/or exercise attenuated hippocampal neuroinflammation and increased BDNF expression ratio in PSD. Notably, NYT, together with exercise therapy, increased BDNF expression and neurogenesis, and reduced proBDNF expression and neuroinflammation in the hippocampus of PSD.

## Conclusions

NYT have multifunctional effects in several diseases, including depression and anxiety. This study revealed that NYT treatment ameliorated serum corticosterone levels in PSD. NYT treatment, together with exercise therapy, improved neurogenesis, the balance of the BDNF/proBDNF ratio, and neuroinflammation in the hippocampus in PSD.

## Supplementary Information

Additional file 1. 3D-HPLC profile of Ninjin’yoeito (TJ-108, Tsumura and Co.). Description of data: This contains the figure file depicting the 3D-HPLC profile of Ninjin’yoeito (TJ-108, Tsumura and Co.).

Additional file 2. Change of feed intake and body weight during experimental periods. Description of data: This contains the figure file depicting the changes of feed intake and body weight during experimental periods.

Additional file 3. Supplementary material for Original underlying images for Western blot. (extension: .pdf).

## Data Availability

The data on which this study is based are available from the corresponding author upon request.

## Abbreviations

NYT: Ninjin’yoeito
PSD: Post-stroke depression
BDNF: Brain-derived neurotrophic factor
proBDNF: Precursor brain-derived neurotrophic factor
DCX: Doublecortin
HPA axis: Hypothalamic-pituitary-adrenal axis
CNS: Central nervous system
mPFC: Medial prefrontal cortex
NF κB: Nuclear factor κB
TrkB: Tropomyosin receptor kinase B
SSRI: Selective serotonin reuptake inhibitor
TrkA: Tropomyosin receptor kinase A
CUMS: Chronic unpredictable mild stress
ET-1: Endothelin-1
Iba-1: Ionized calcium-binding adapter molecule-1
GFAP: Glial fibrillary acidic protein
DG: Dentate gyrus
TNF-α: Tumor necrosis factor-α
iNOS: inducible Nitric oxide synthase
