# Supplementary figures and images for: Effects of Ninjin’yoeito and physical exercise on serum corticosterone and hippocampal BDNF/proBDNF and neuroinflammation in post-stroke depression in rats
Source: BMC Complement Med Ther. 2025 May 13;25:171. doi: 10.1186/s12906-025-04915-w (PMC12070628; doi:10.1186/s12906-025-04915-w)

## Slide 1
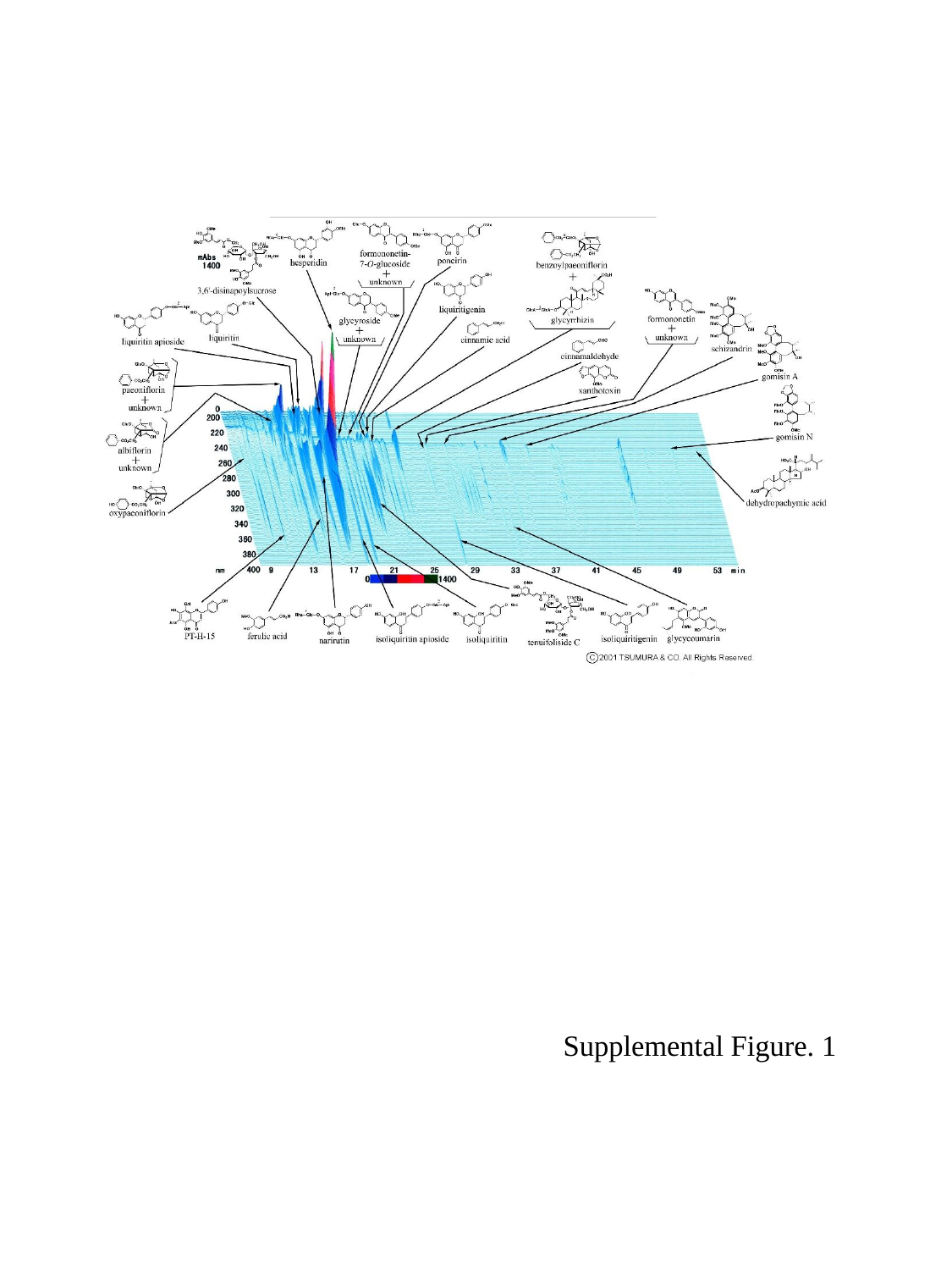

Supplemental Figure. 1

Supplement: Supplementary file 1 — Additional file 1. 3D-HPLC profile of Ninjin’yoeito (TJ-108, Tsumura and Co.). Description of data: This contains the figure file depicting the 3D-HPLC profile of Ninjin’yoeito (TJ-108, Tsumura and Co.). [file 12906_2025_4915_MOESM1_ESM.pptx]

## Slide 1
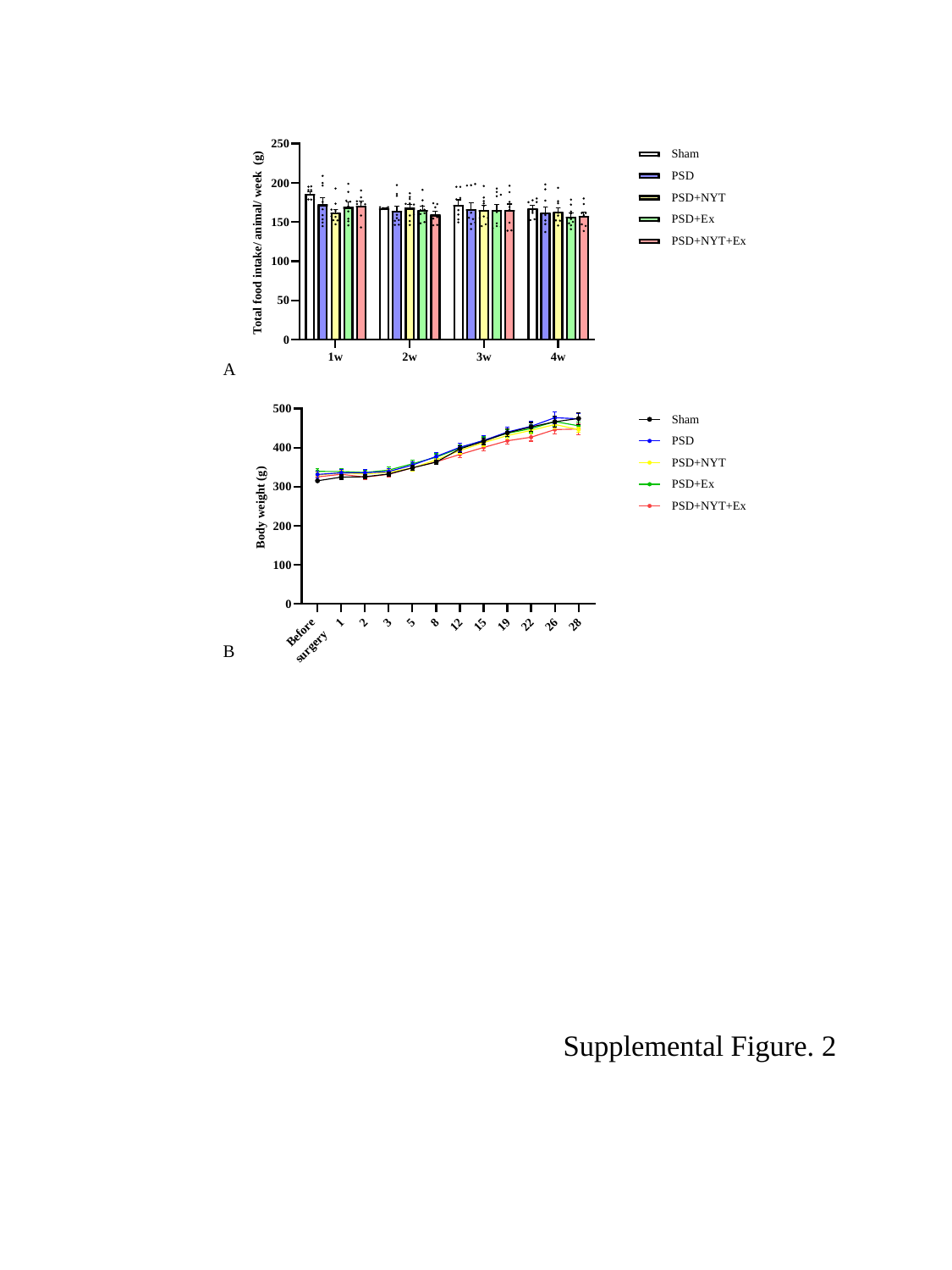

A
B
Supplemental Figure. 2

Supplement: Supplementary file 2 — Additional file 2. Change of feed intake and body weight during experimental periods. Description of data: This contains the figure file depicting the changes of feed intake and body weight during experimental periods. [file 12906_2025_4915_MOESM2_ESM.pptx]
