# Supplementary material for: Effects of Ninjin’yoeito and physical exercise on serum corticosterone and hippocampal BDNF/proBDNF and neuroinflammation in post-stroke depression in rats
Source: BMC Complement Med Ther. 2025 May 13;25:171. doi: 10.1186/s12906-025-04915-w (PMC12070628; doi:10.1186/s12906-025-04915-w)

Supplementary material for Original underlying images for Western blot.

Figure 3

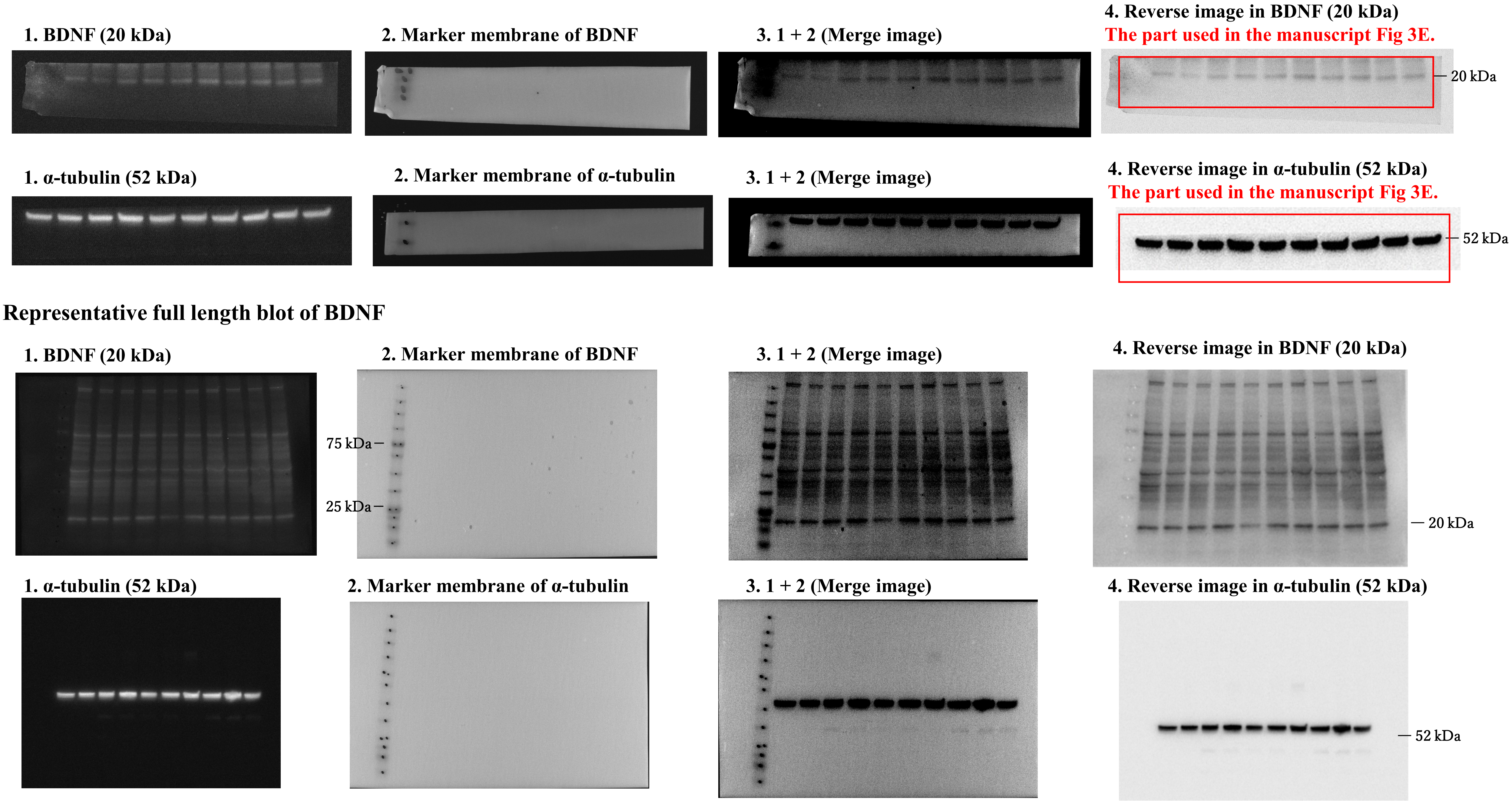
1. BDNF (20 kDa)
2. Marker membrane of BDNF
3. 1 + 2 (Merge image)
4. Reverse image in BDNF (20 kDa)
The part used in the manuscript Fig 3E.
20 kDa
1. α-tubulin (52 kDa)
2. Marker membrane of α-tubulin
3. 1 + 2 (Merge image)
4. Reverse image in α-tubulin (52 kDa)
The part used in the manuscript Fig 3E.
52 kDa
Representative full length blot of BDNF
1. BDNF (20 kDa)
2. Marker membrane of BDNF
75 kDa
25 kDa
3. 1 + 2 (Merge image)
4. Reverse image in BDNF (20 kDa)
20 kDa
1. α-tubulin (52 kDa)
2. Marker membrane of α-tubulin
3. 1 + 2 (Merge image)
4. Reverse image in α-tubulin (52 kDa)
52 kDa

Figure 3

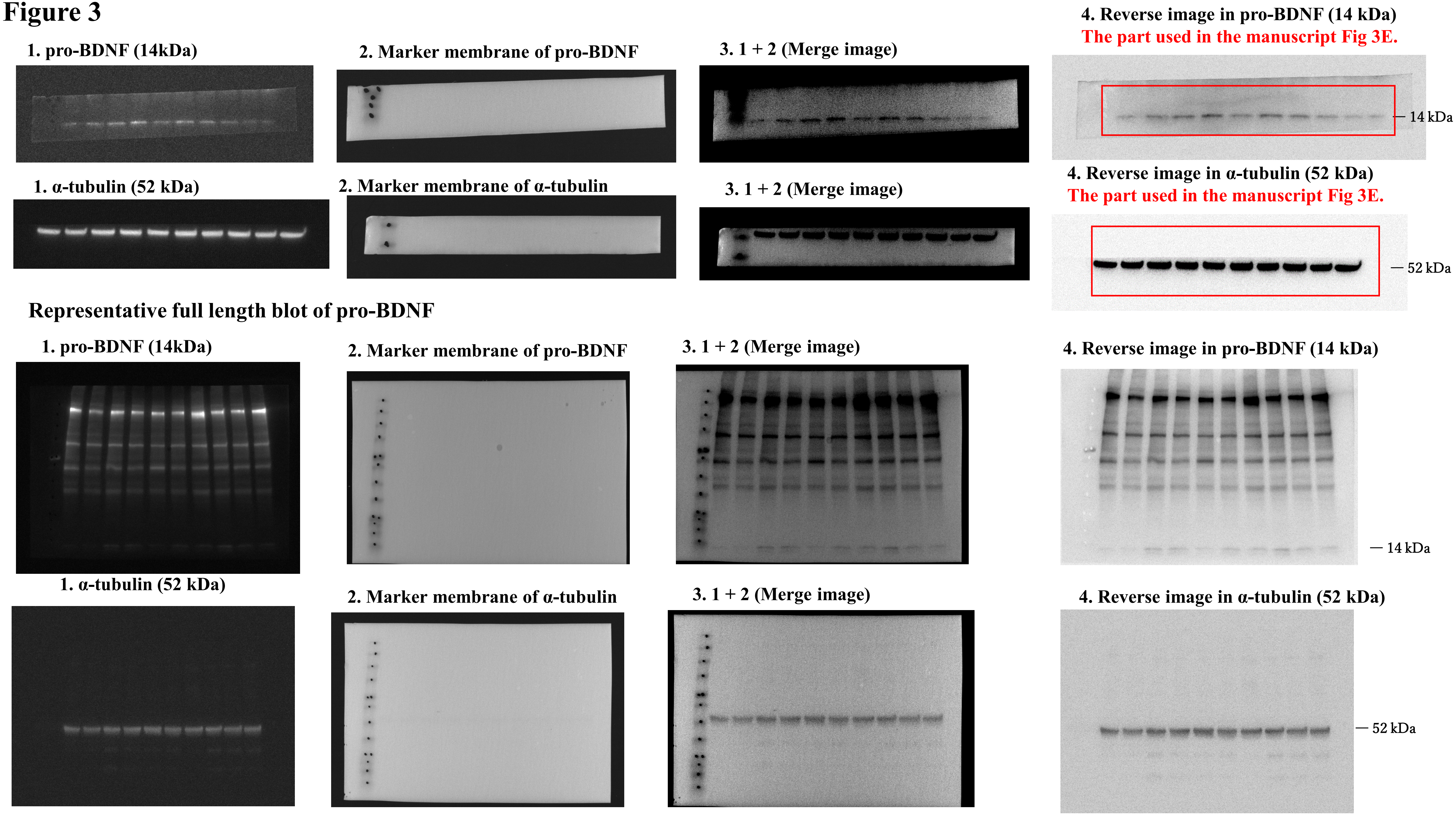
1. pro-BDNF (14kDa)
2. Marker membrane of pro-BDNF
3. 1 + 2 (Merge image)
4. Reverse image in pro-BDNF (14 kDa)
The part used in the manuscript Fig 3E.
14 kDa
1. α-tubulin (52 kDa)
2. Marker membrane of α-tubulin
3. 1 + 2 (Merge image)
4. Reverse image in α-tubulin (52 kDa)
The part used in the manuscript Fig 3E.
52 kDa
Representative full length blot of pro-BDNF
1. pro-BDNF (14kDa)
2. Marker membrane of pro-BDNF
3. 1 + 2 (Merge image)
4. Reverse image in pro-BDNF (14 kDa)
14 kDa
1. α-tubulin (52 kDa)
2. Marker membrane of α-tubulin
3. 1 + 2 (Merge image)
4. Reverse image in α-tubulin (52 kDa)
52 kDa

Figure 4

1. NeuN (50 kDa)

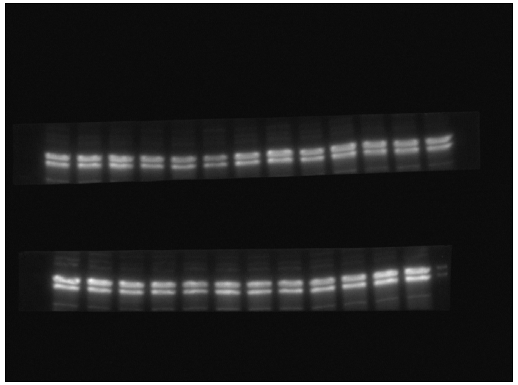

2. Marker membrane of NeuN

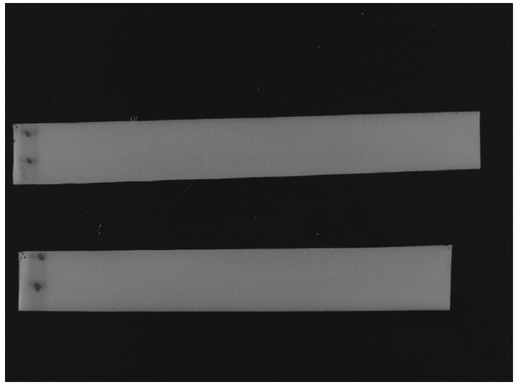

3. 1 + 2 (Merge image)

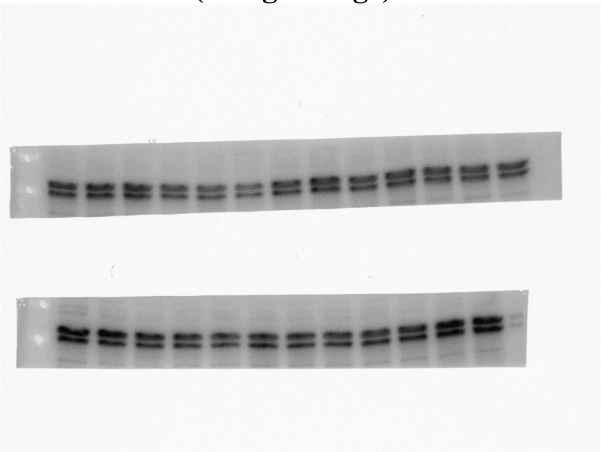

4. Reverse image in NeuN (50 kDa)

The upper part used in the manuscript Fig 4C.

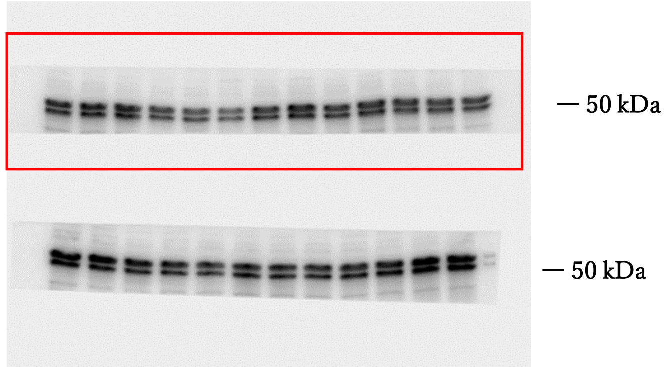

1. α-tubulin (52 kDa)

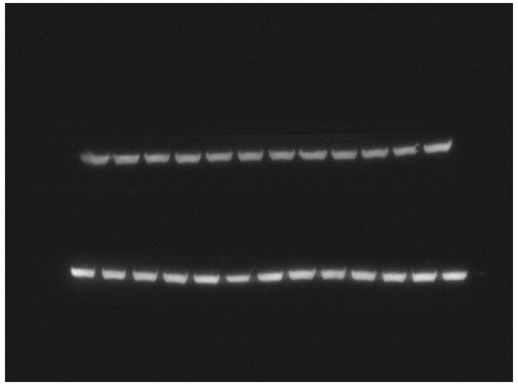

2. Marker membrane of α-tubulin

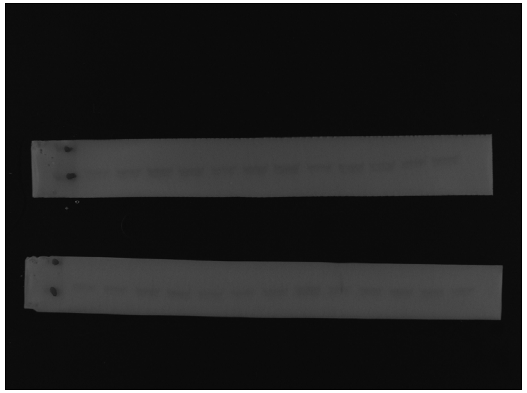

3. 1 + 2 (Merge image)

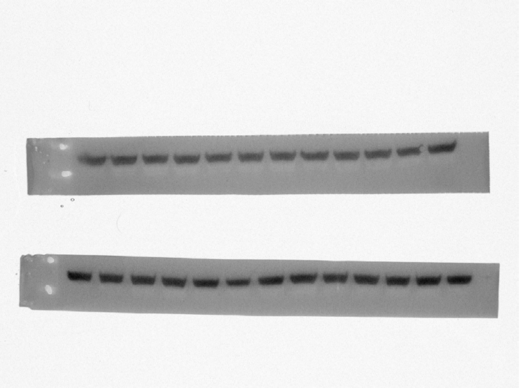

4. Reverse image in α-tubulin (52 kDa)

The lower part used in the manuscript Fig 4C.

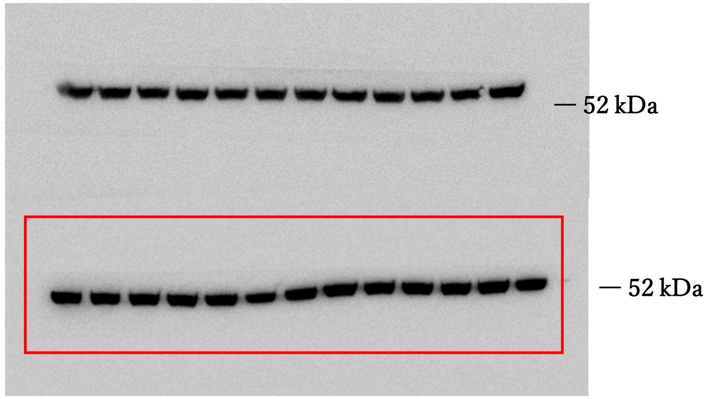

Figure 5

First membrane

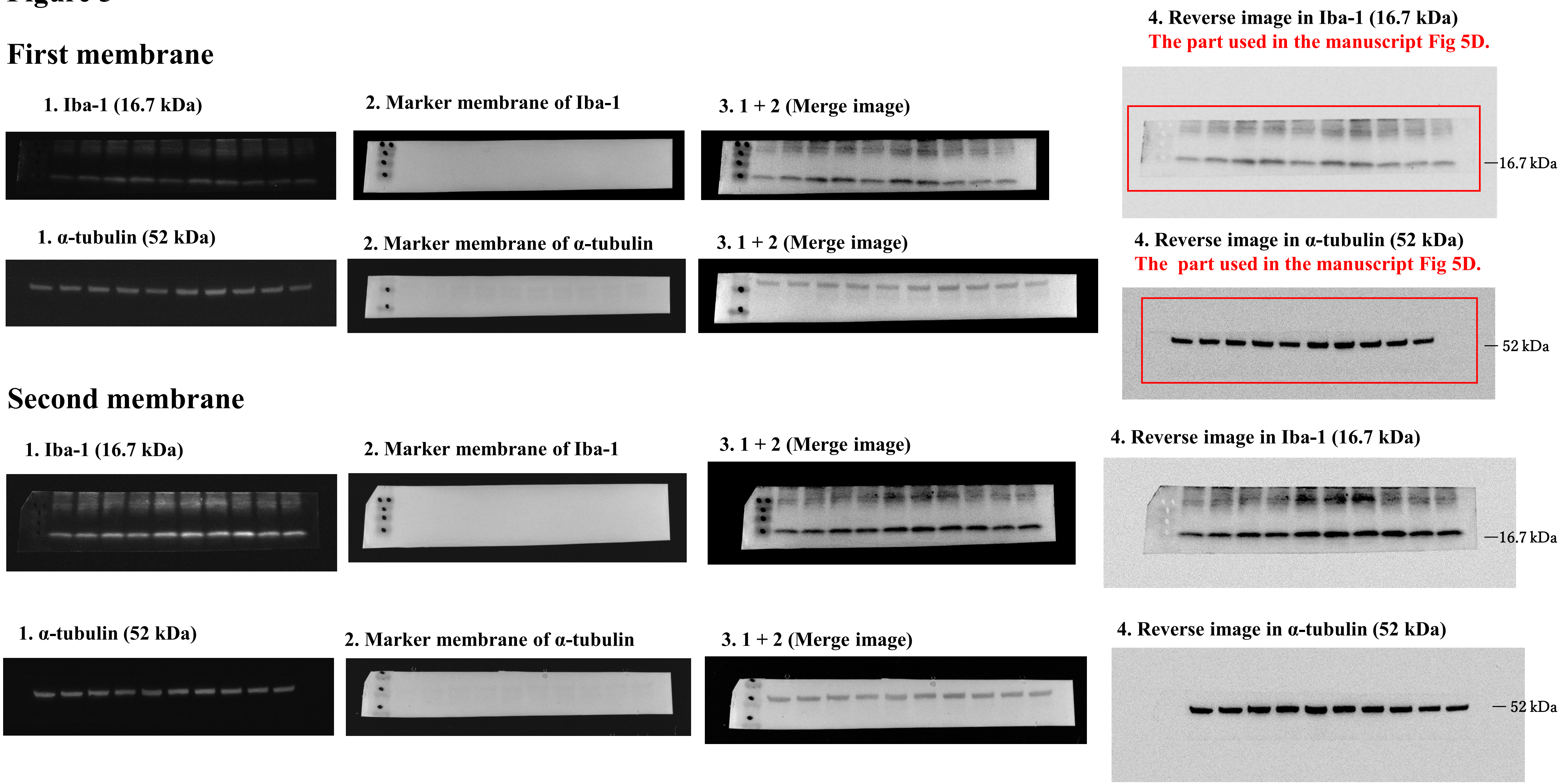

Figure 5

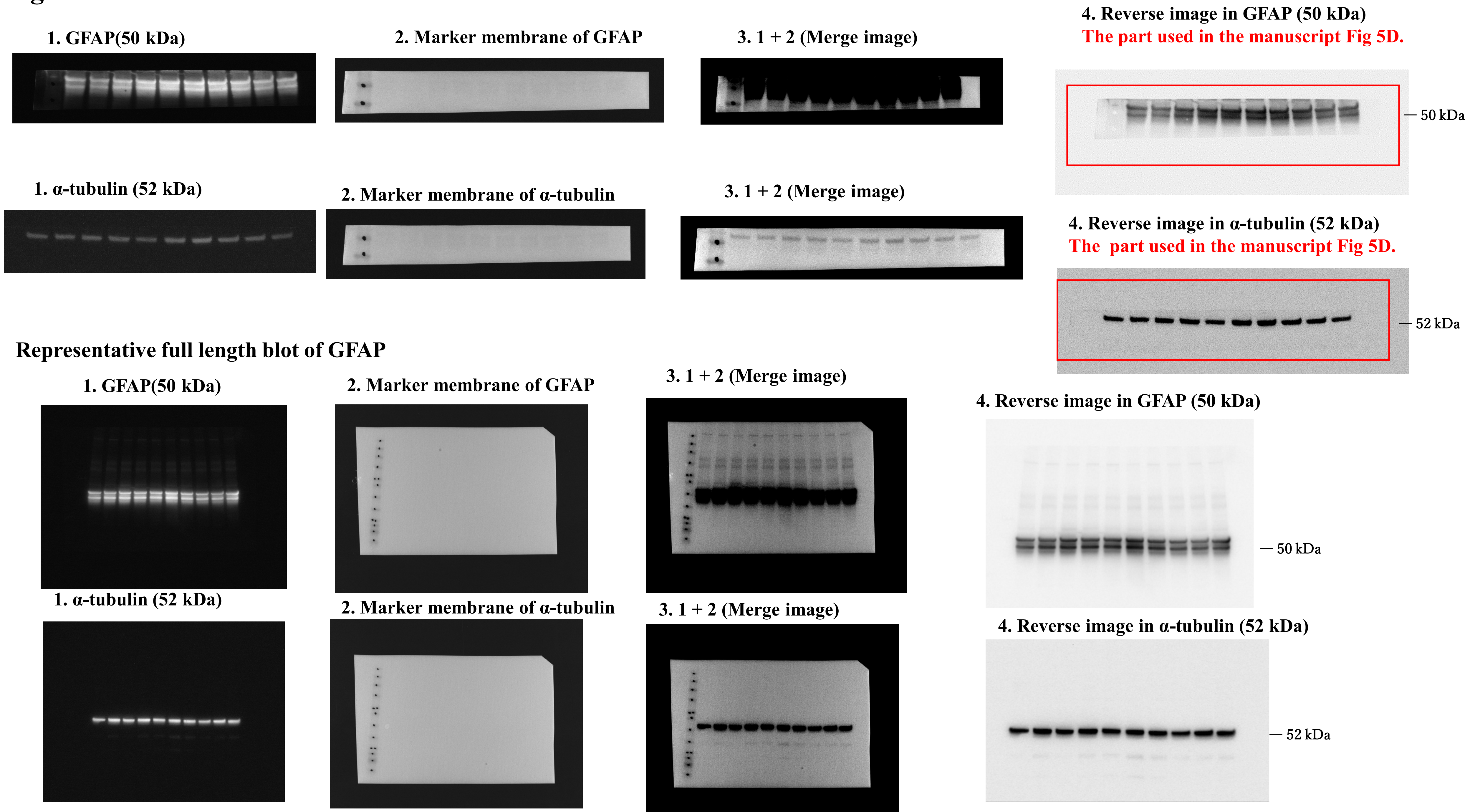

1. GFAP(50 kDa)
2. Marker membrane of GFAP
3. 1 + 2 (Merge image)
4. Reverse image in GFAP (50 kDa)
The part used in the manuscript Fig 5D.
50 kDa
1. α-tubulin (52 kDa)
2. Marker membrane of α-tubulin
3. 1 + 2 (Merge image)
4. Reverse image in α-tubulin (52 kDa)
The part used in the manuscript Fig 5D.
52 kDa
Representative full length blot of GFAP
1. GFAP(50 kDa)
2. Marker membrane of GFAP
3. 1 + 2 (Merge image)
4. Reverse image in GFAP (50 kDa)
50 kDa
1. α-tubulin (52 kDa)
2. Marker membrane of α-tubulin
3. 1 + 2 (Merge image)
4. Reverse image in α-tubulin (52 kDa)
52 kDa

Figure 5

First membrane

1. TNF-α (26 kDa)

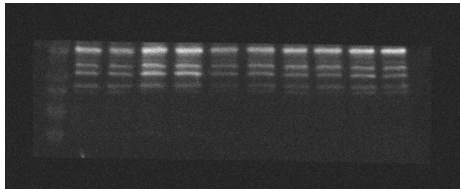

2. Marker membrane of TNF-α

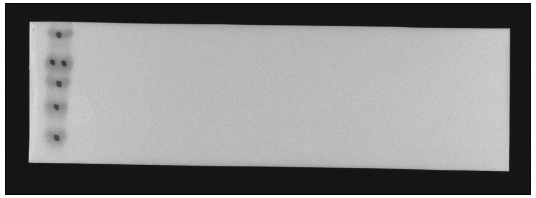

3. 1 + 2 (Merge image)

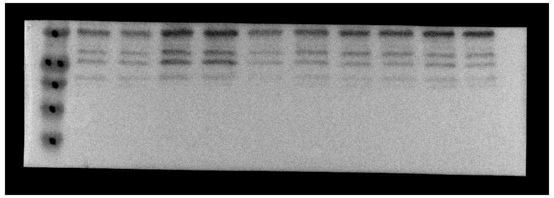

4. Reverse image in TNF-α (26 kDa)

The part used in the manuscript Fig 5E.

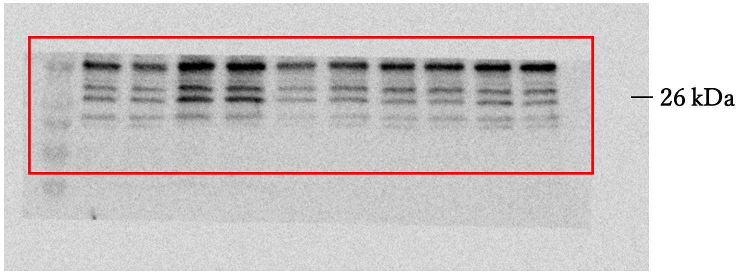

1. α-tubulin (52 kDa)

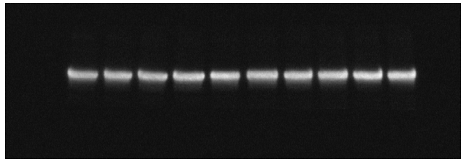

2. Marker membrane of α-tubulin

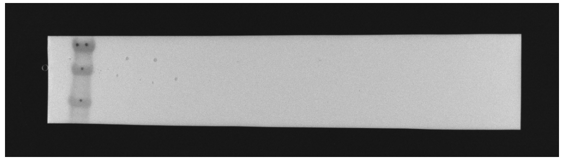

3. 1 + 2 (Merge image)

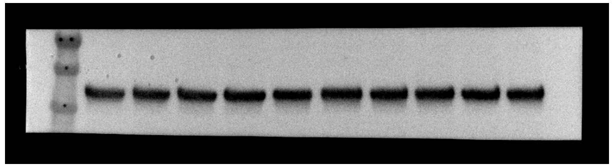

4. Reverse image in α-tubulin (52 kDa)

The part used in the manuscript Fig 5E.

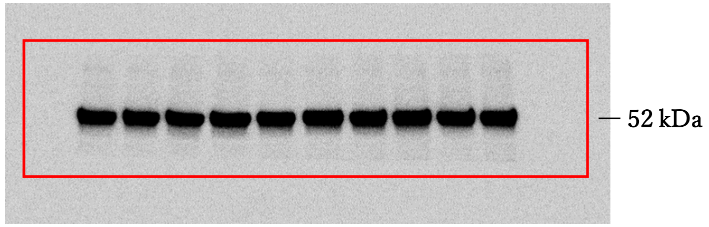

Second membrane

1. TNF-α (26 kDa)

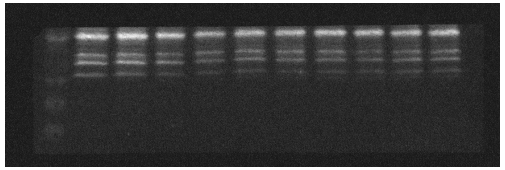

2. Marker membrane of TNF-α

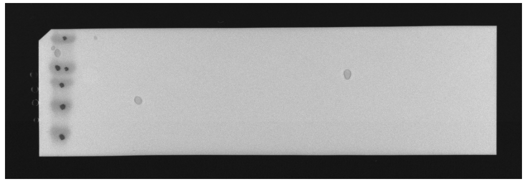

3. 1 + 2 (Merge image)

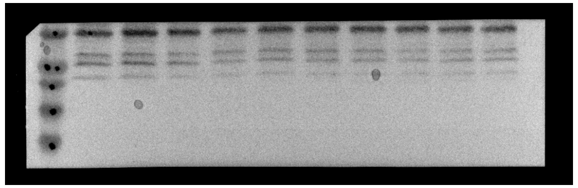

4. Reverse image in TNF-α (26 kDa)

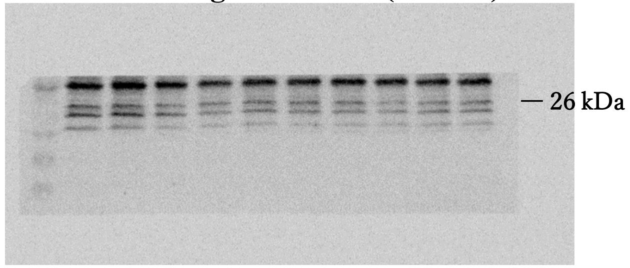

1. α-tubulin (52 kDa)

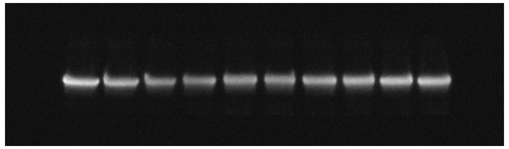

2. Marker membrane of α-tubulin

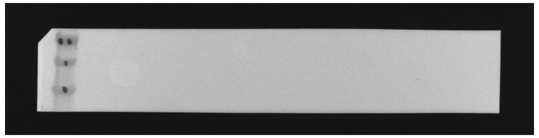

3. 1 + 2 (Merge image)

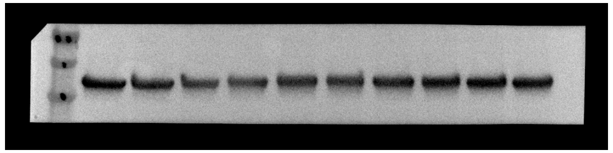

4. Reverse image in α-tubulin (52 kDa)

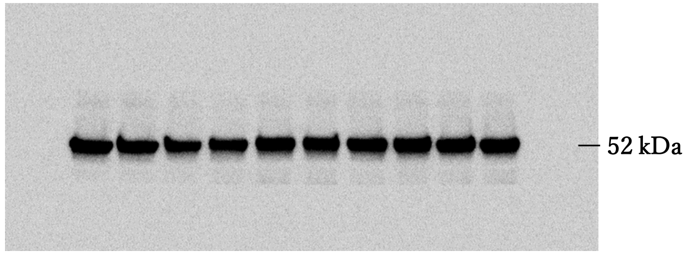

Supplement: Supplementary file 3 — Additional file 3. Supplementary material for Original underlying images for Western blot. (extension: .pdf). [file 12906_2025_4915_MOESM3_ESM.pdf]
